# (*S*)-5-(*l*-Menth­yloxy)-5-[(2*R*,3*R*)-2-(*l*-menth­yloxy)-5-oxotetra­hydro­furan-3-yl]furan-2(5*H*)-one

**DOI:** 10.1107/S1600536809053690

**Published:** 2009-12-19

**Authors:** Bing-Bing Zhang, Yu-Qin Fu, Jing-Chao Tao

**Affiliations:** a Department of Chemistry, Zhengzhon University, Zhengzhou 450052, People’s Republic of China; b College of Chemistry and Chemical Engineering, Luoyang Normal University, Luoyang 471022, People’s Republic of China

## Abstract

In the title compound, C_28_H_44_O_6_, the two five-membered rings form a dihedral angle of 6.7 (1)°. In the crystal structure, weak inter­molecular C—H⋯O hydrogen bonds link mol­ecules into layers parallel to (101).

## Related literature

For the applications of 5-(*R*)-(*l*-menth­yloxy)-2(5*H*)-furan­one in asymmetic synthesis, see: Huang & Chen (1999 ▶); Wang & Chen (1999 ▶); Fu *et al.* (2003 ▶); Yu *et al.* (2008 ▶).
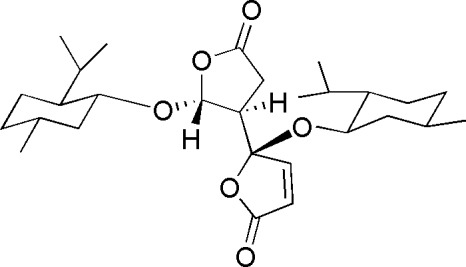


## Experimental

### 

#### Crystal data

C_28_H_44_O_6_
*M* *_r_* = 476.63
Monoclinic, 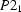

*a* = 12.3443 (15) Å
*b* = 9.3455 (11) Å
*c* = 12.5044 (15) Å
β = 92.990 (2)°
*V* = 1440.6 (3) Å^3^
*Z* = 2
Mo *K*α radiation
μ = 0.08 mm^−1^
*T* = 296 K
0.37 × 0.21 × 0.13 mm

#### Data collection

Bruker SMART APEXII CCD area-detector diffractometer
Absorption correction: multi-scan (*SADABS*; Sheldrick, 1996 ▶) *T*_min_ = 0.973, *T*_max_ = 0.990
10978 measured reflections
2847 independent reflections
1948 reflections with *I* > 2σ(*I*)
*R*_int_ = 0.037

#### Refinement

*R*[*F* ^2^ > 2σ(*F* ^2^)] = 0.041
*wR*(*F* ^2^) = 0.107
*S* = 1.10
2847 reflections
313 parameters
1 restraint
H-atom parameters constrained
Δρ_max_ = 0.13 e Å^−3^
Δρ_min_ = −0.11 e Å^−3^

### 

Data collection: *SMART* (Bruker, 2004 ▶); cell refinement: *SAINT* (Bruker, 2004 ▶); data reduction: *SAINT*; program(s) used to solve structure: *SHELXS97* (Sheldrick, 2008 ▶); program(s) used to refine structure: *SHELXL97* (Sheldrick, 2008 ▶); molecular graphics: *SHELXTL* (Sheldrick, 2008 ▶); software used to prepare material for publication: *SHELXTL* and *PLATON* (Spek, 2009 ▶).

## Supplementary Material

Crystal structure: contains datablocks global, I. DOI: 10.1107/S1600536809053690/cv2674sup1.cif

Structure factors: contains datablocks I. DOI: 10.1107/S1600536809053690/cv2674Isup2.hkl

Additional supplementary materials:  crystallographic information; 3D view; checkCIF report

## Figures and Tables

**Table 1:** Hydrogen-bond geometry (Å, °)

*D*—H⋯*A*	*D*—H	H⋯*A*	*D*⋯*A*	*D*—H⋯*A*
C3—H3⋯O5^i^	0.93	2.37	3.289 (5)	168
C28—H28*A*⋯O2^ii^	0.96	2.59	3.405 (5)	143

Symmetry codes: (i) 
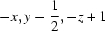
; (ii) 
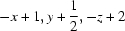
.

**Fig. 1.**
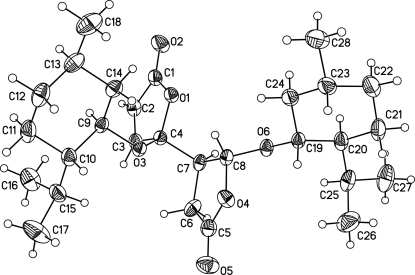
The molecular structure of (I) showing the atomic numbering and 20% probability displacement ellipsoids.

**Fig. 2.**
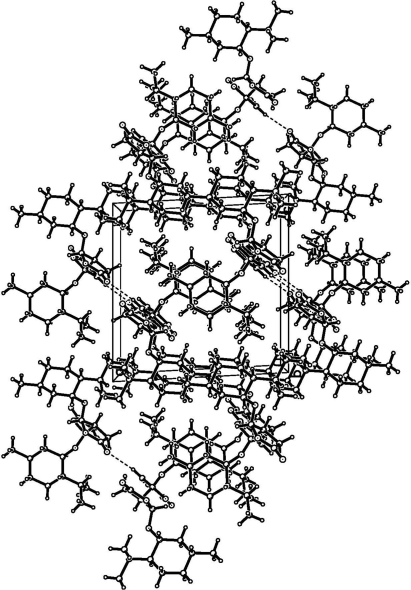
A portion of the crystal packing showing weak intermolecular C—H···O hydrogen-bonds as dashed lines.

## supplementary crystallographic information

### Comment

The well known chiral 5-(*R*)-(*l*-menthyloxy)-2(5*H*)-furanone behaves as a Michael acceptor towards carbon, oxygen, sulfur and nitrogen nucleophiles to afford chiral 4-(*R*)-(*l*-menthyloxy)-3-substituted-butyrolactone (Huang *et al.*, 1999; Wang *et al.*, 1999; Fu *et al.*, 2003; Yu *et al.*, 2008). Recently, when we have used 1,2:5,6-di-*O*-isopropylidene-*D*-glucofuranose as Michael donor to react with 5-(*R*)-(*l*-menthyloxy)-2(5*H*)-furanone, the adduct was not yielded, but the title compound, (I) - unexpected product of the self-addition of 5-(*R*)-(*l*-menthyloxy)-2(5*H*)-furanone - was obtained. Herein, we report the synthesis, characterization and crystal structure of (I) (Fig. 1).

In the crystal structure, each molecule is connected by six adjacent molecules through weak intermolecular C—H···O hydrogen bonds (Table 1) between H atoms of menthyloxy groups, or H atoms of lactone, and O atoms of the carbonyl groups, leading to the formation of a two-dimensional sheet parallel to (*a+c*)*b* plane (Fig. 2). The layers are further packed through van der Waals forces..

The absolute configuration of the title compound was established on the basis of the chiral *l*-menthyloxy group. In the addition reaction, the three chiral centers on the *l*-menthyloxy group did not change because they did not participant in the reaction. Accordingly, the stereogenic center of C7 is determined as *R*, the configuration of C4 and C8 are retained.

### Experimental

1,2:5,6-di-*O*-isopropylidene-α-*D*-glucofuranose (2 mmol, 0.520 g) was added to the mixture of powdered K_2_CO_3_ (10 mmol, 1.382 g), tetrabutylammonium bromide (2 mmol, 0.645 g) and acetonitrile (20 ml). The mixture was stirred for 20 minutes, then the chiral synthon 5-(*R*)-(*l*-menthyloxy)-2(5*H*)-furanone (4 mmol, 0.953 g) was added and the mixture was stirred at room temperature for 8 days until TLC analysis indicated that the chiral synthon had been completely consumed. After the addition of acetonitrile (50 ml), the mixture was filtered and the salts were washed with acetonitrile. The organic layer was dried over MgSO_4_, evaporated, and purified by column chromatography to give a white solid, which was recrystalized to afford colorless crystal.

Yield 38.8%, m.p. 143–144 °C, [α]_D_^20^: -159 (c 0.1, CHCl_3_); IR (KBr, cm^-1^): 3096,3079, 2957, 2918, 2872, 1810, 1781, 1768, 1612, 1456, 1384, 1369, 905; ^1^HNMR (400 MHz, CDCl_3_) δ: 0.68 (d, J = 6.8 Hz, 3H, CH_3_,), 0.76 (d, J = 6.8 Hz, 3H, CH_3_), 0.83 (d, J = 6.4 Hz, 3H, CH_3_,), 0.86 (d, J = 6.4 Hz, 3H,CH_3_), 0.91 (d, J = 6.8 Hz, 3H, CH_3_), 0.93 (d, J = 6.4 Hz, 3H, CH_3_),1.00–1.19 (m, 6H, 6CH), 1.25–1.65 (m, 8H, 4CH_2_), 2.03–2.05 (m, 4H, 2CH_2_), 2.45 (m, 1H, CH), 2.75–2.82 (m, 2H, 2CH), 3.17–3.20 (m,1*H*, CH), 3.50–3.53 (m, 1H, CH), 5.65 (d, J = 1.6 Hz, 1H, OCH), 6.36 (d, J = 5.6 Hz, 1H, CH), 7.02 (d, J = 5.6 Hz, 1H, CH); ^13^C NMR (100 MHz, CDCl_3_) δ: 15.6, 15.8, 20.9, 21.2,22.1, 22.3, 22.7, 23.1, 25.3, 25.5, 29.5, 31.4, 31.5, 33.9, 34.3, 39.6, 43.4,47.7, 48.2, 49.4, 75.8, 77.4, 100.4, 110.0, 126.6, 150.9, 168.5, 174.4; HRMS (ESI) m/*z*: calcd. for C_28_H_44_O_6_ (*M*+Na^+^) 499.3036, found 499.3035.

### Refinement

All H atoms were geometrically positioned (C—H 0.93-0.98 Å) and refined as riding, with *U*_iso_(H) = 1.2-1.5 *U*_eq_(C). In the absence of any significant anomalous scatterers in the molecule, the 2428 Friedel pairs were merged before the final refinement.

### Crystal data

**Table:**

C_28_H_44_O_6_	*F*(000) = 520
*M**_r_* = 476.63	*D*_x_ = 1.099 Mg m^−^^3^
Monoclinic, *P*2_1_	Melting point: 416 K
Hall symbol: P 2y1	Mo *K*α radiation, λ = 0.71073 Å
*a* = 12.3443 (15) Å	Cell parameters from 2076 reflections
*b* = 9.3455 (11) Å	θ = 2.4–19.9°
*c* = 12.5044 (15) Å	µ = 0.08 mm^−^^1^
β = 92.990 (2)°	*T* = 296 K
*V* = 1440.6 (3) Å^3^	Block, colourless
*Z* = 2	0.37 × 0.21 × 0.13 mm

### Data collection

**Table:**

Bruker SMART APEXII CCD area-detector diffractometer	2847 independent reflections
Radiation source: fine-focus sealed tube	1948 reflections with *I* > 2σ(*I*)
graphite	*R*_int_ = 0.037
φ and ω scans	θ_max_ = 25.5°, θ_min_ = 2.4°
Absorption correction: multi-scan (*SADABS*; Sheldrick, 1996)	*h* = −14→14
*T*_min_ = 0.973, *T*_max_ = 0.990	*k* = −11→11
10978 measured reflections	*l* = −15→14

### Refinement

**Table:**

Refinement on *F*^2^	Primary atom site location: structure-invariant direct methods
Least-squares matrix: full	Secondary atom site location: difference Fourier map
*R*[*F*^2^ > 2σ(*F*^2^)] = 0.041	Hydrogen site location: inferred from neighbouring sites
*wR*(*F*^2^) = 0.107	H-atom parameters constrained
*S* = 1.10	*w* = 1/[σ^2^(*F*_o_^2^) + (0.0511*P*)^2^ + 0.0145*P*] where *P* = (*F*_o_^2^ + 2*F*_c_^2^)/3
2847 reflections	(Δ/σ)_max_ < 0.001
313 parameters	Δρ_max_ = 0.13 e Å^−^^3^
1 restraint	Δρ_min_ = −0.11 e Å^−^^3^

### Special details

**Table:**

Geometry. All e.s.d.'s (except the e.s.d. in the dihedral angle between two l.s. planes)are estimated using the full covariance matrix. The cell e.s.d.'s are takeninto account individually in the estimation of e.s.d.'s in distances, anglesand torsion angles; correlations between e.s.d.'s in cell parameters are onlyused when they are defined by crystal symmetry. An approximate (isotropic)treatment of cell e.s.d.'s is used for estimating e.s.d.'s involving l.s. planes.
Refinement. Refinement of *F*^2^ against ALL reflections. The weighted *R*-factor *wR* and goodness of fit *S* are based on *F*^2^, conventional *R*-factors *R* are based on *F*, with *F* set to zero for negative *F*^2^. The threshold expression of *F* > 2 σ (*F*^2^) is used only for calculating *R*-factors(gt) *etc*. and is not relevant to the choice of reflections for refinement. *R*-factors based on *F*^2^ are statistically about twice as large as those based on *F*, and *R*-factors based on ALL data will be even larger.

### Fractional atomic coordinates and isotropic or equivalent isotropic displacement parameters (Å^2^)

**Table:**

	*x*	*y*	*z*	*U*_iso_*/*U*_eq_	
C1	0.2722 (3)	−0.1115 (4)	0.7313 (3)	0.0636 (9)	
C2	0.1833 (3)	−0.1464 (4)	0.6548 (3)	0.0673 (9)	
H2	0.1539	−0.2371	0.6435	0.081*	
C3	0.1512 (3)	−0.0294 (3)	0.6040 (2)	0.0580 (8)	
H3	0.0966	−0.0252	0.5499	0.070*	
C4	0.2157 (2)	0.0963 (3)	0.6464 (2)	0.0500 (7)	
C5	0.0993 (3)	0.4327 (4)	0.6196 (3)	0.0668 (9)	
C6	0.0638 (3)	0.2810 (4)	0.6295 (3)	0.0721 (10)	
H6A	0.0590	0.2349	0.5599	0.087*	
H6B	−0.0066	0.2761	0.6603	0.087*	
C7	0.1496 (2)	0.2092 (3)	0.7026 (2)	0.0542 (8)	
H7	0.1145	0.1645	0.7627	0.065*	
C8	0.2212 (3)	0.3329 (3)	0.7451 (2)	0.0542 (8)	
H8	0.2980	0.3106	0.7377	0.065*	
C9	0.3545 (2)	0.0949 (3)	0.5128 (2)	0.0516 (7)	
H9	0.3389	−0.0079	0.5119	0.062*	
C10	0.3496 (3)	0.1518 (4)	0.3983 (2)	0.0639 (9)	
H10	0.3613	0.2553	0.4040	0.077*	
C11	0.4467 (3)	0.0921 (5)	0.3399 (3)	0.0882 (12)	
H11A	0.4386	−0.0107	0.3321	0.106*	
H11B	0.4466	0.1334	0.2687	0.106*	
C12	0.5536 (3)	0.1242 (5)	0.3987 (3)	0.0906 (13)	
H12A	0.6118	0.0834	0.3593	0.109*	
H12B	0.5641	0.2270	0.4016	0.109*	
C13	0.5596 (3)	0.0655 (4)	0.5105 (3)	0.0786 (11)	
H13	0.5532	−0.0389	0.5056	0.094*	
C14	0.4636 (3)	0.1210 (4)	0.5701 (3)	0.0630 (9)	
H14A	0.4642	0.0756	0.6399	0.076*	
H14B	0.4726	0.2231	0.5816	0.076*	
C15	0.2398 (3)	0.1322 (4)	0.3384 (3)	0.0806 (11)	
H15	0.1842	0.1611	0.3872	0.097*	
C16	0.2143 (4)	−0.0210 (5)	0.3039 (3)	0.1099 (15)	
H16A	0.1450	−0.0238	0.2653	0.165*	
H16B	0.2125	−0.0811	0.3660	0.165*	
H16C	0.2693	−0.0547	0.2585	0.165*	
C17	0.2291 (5)	0.2304 (7)	0.2419 (4)	0.146 (2)	
H17A	0.2808	0.2030	0.1910	0.219*	
H17B	0.2425	0.3273	0.2642	0.219*	
H17C	0.1571	0.2229	0.2094	0.219*	
C18	0.6660 (3)	0.0997 (6)	0.5710 (4)	0.1129 (16)	
H18A	0.7251	0.0630	0.5322	0.169*	
H18B	0.6670	0.0563	0.6406	0.169*	
H18C	0.6734	0.2015	0.5785	0.169*	
C19	0.2668 (3)	0.4725 (3)	0.9018 (2)	0.0555 (8)	
H19	0.2633	0.5571	0.8555	0.067*	
C20	0.2145 (3)	0.5078 (4)	1.0059 (2)	0.0703 (10)	
H20	0.2177	0.4204	1.0492	0.084*	
C21	0.2846 (4)	0.6188 (4)	1.0667 (3)	0.0892 (12)	
H21A	0.2800	0.7087	1.0279	0.107*	
H21B	0.2555	0.6344	1.1363	0.107*	
C22	0.4031 (4)	0.5765 (5)	1.0825 (3)	0.0920 (12)	
H22A	0.4432	0.6540	1.1177	0.110*	
H22B	0.4092	0.4931	1.1285	0.110*	
C23	0.4521 (3)	0.5434 (4)	0.9774 (3)	0.0752 (10)	
H23	0.4483	0.6301	0.9332	0.090*	
C24	0.3841 (3)	0.4271 (4)	0.9195 (3)	0.0690 (9)	
H24A	0.4140	0.4070	0.8509	0.083*	
H24B	0.3877	0.3399	0.9616	0.083*	
C25	0.0957 (3)	0.5478 (5)	0.9905 (3)	0.0878 (12)	
H25	0.0598	0.4668	0.9538	0.105*	
C26	0.0741 (4)	0.6783 (6)	0.9189 (4)	0.1279 (18)	
H26A	0.1058	0.7616	0.9528	0.192*	
H26B	−0.0028	0.6920	0.9077	0.192*	
H26C	0.1056	0.6632	0.8512	0.192*	
C27	0.0408 (4)	0.5658 (7)	1.0960 (4)	0.136 (2)	
H27A	0.0563	0.4842	1.1409	0.204*	
H27B	−0.0362	0.5739	1.0822	0.204*	
H27C	0.0676	0.6508	1.1315	0.204*	
C28	0.5722 (3)	0.4972 (5)	0.9912 (4)	0.1014 (14)	
H28A	0.5779	0.4139	1.0361	0.152*	
H28B	0.6142	0.5736	1.0237	0.152*	
H28C	0.5990	0.4754	0.9224	0.152*	
O1	0.29140 (17)	0.0331 (2)	0.72584 (15)	0.0571 (5)	
O2	0.3238 (3)	−0.1869 (3)	0.7920 (2)	0.0953 (9)	
O3	0.27166 (15)	0.16972 (19)	0.56934 (14)	0.0511 (5)	
O4	0.18960 (19)	0.4565 (2)	0.68011 (17)	0.0668 (6)	
O5	0.0572 (2)	0.5251 (3)	0.5659 (2)	0.0991 (9)	
O6	0.20131 (17)	0.3581 (2)	0.85066 (15)	0.0589 (6)	

### Atomic displacement parameters (Å^2^)

**Table:**

	*U*^11^	*U*^22^	*U*^33^	*U*^12^	*U*^13^	*U*^23^
C1	0.097 (3)	0.046 (2)	0.0488 (19)	0.0005 (19)	0.0076 (19)	0.0070 (16)
C2	0.097 (3)	0.0419 (19)	0.064 (2)	−0.0128 (19)	0.0108 (19)	−0.0021 (17)
C3	0.072 (2)	0.0488 (19)	0.0540 (17)	−0.0082 (17)	0.0060 (15)	−0.0059 (16)
C4	0.0595 (18)	0.0457 (17)	0.0455 (16)	−0.0009 (15)	0.0078 (15)	0.0032 (14)
C5	0.071 (2)	0.061 (2)	0.069 (2)	−0.0050 (19)	0.006 (2)	−0.0044 (19)
C6	0.059 (2)	0.053 (2)	0.105 (3)	0.0005 (17)	0.0085 (19)	−0.014 (2)
C7	0.062 (2)	0.0470 (17)	0.0554 (18)	−0.0062 (16)	0.0189 (16)	−0.0048 (15)
C8	0.0646 (19)	0.0480 (18)	0.0511 (17)	0.0001 (15)	0.0126 (14)	−0.0054 (15)
C9	0.067 (2)	0.0361 (15)	0.0534 (17)	0.0045 (15)	0.0177 (15)	0.0029 (14)
C10	0.084 (2)	0.055 (2)	0.0536 (18)	−0.0023 (18)	0.0141 (17)	0.0020 (16)
C11	0.106 (3)	0.094 (3)	0.068 (2)	0.001 (3)	0.036 (2)	−0.006 (2)
C12	0.091 (3)	0.084 (3)	0.100 (3)	−0.003 (2)	0.045 (2)	−0.009 (3)
C13	0.072 (2)	0.050 (2)	0.116 (3)	0.0031 (18)	0.019 (2)	−0.003 (2)
C14	0.067 (2)	0.0487 (19)	0.074 (2)	0.0003 (17)	0.0115 (18)	0.0031 (17)
C15	0.102 (3)	0.085 (3)	0.0549 (19)	0.001 (2)	0.005 (2)	0.010 (2)
C16	0.129 (4)	0.106 (4)	0.094 (3)	−0.033 (3)	0.002 (3)	−0.002 (3)
C17	0.194 (6)	0.141 (5)	0.098 (3)	−0.025 (5)	−0.035 (4)	0.046 (3)
C18	0.065 (3)	0.104 (4)	0.171 (5)	0.004 (3)	0.014 (3)	−0.008 (3)
C19	0.081 (2)	0.0380 (16)	0.0480 (16)	−0.0005 (16)	0.0063 (15)	−0.0009 (14)
C20	0.108 (3)	0.054 (2)	0.0495 (18)	−0.003 (2)	0.0151 (18)	−0.0012 (16)
C21	0.133 (4)	0.071 (3)	0.064 (2)	0.000 (3)	0.015 (2)	−0.018 (2)
C22	0.134 (4)	0.075 (3)	0.065 (2)	−0.011 (3)	−0.012 (2)	−0.002 (2)
C23	0.095 (3)	0.060 (2)	0.069 (2)	0.002 (2)	−0.0137 (19)	0.0050 (19)
C24	0.089 (3)	0.055 (2)	0.062 (2)	0.0030 (19)	−0.0032 (18)	0.0035 (17)
C25	0.106 (3)	0.077 (3)	0.084 (3)	0.002 (2)	0.035 (2)	−0.020 (2)
C26	0.108 (4)	0.135 (5)	0.143 (4)	0.044 (3)	0.026 (3)	0.002 (4)
C27	0.156 (4)	0.141 (5)	0.118 (4)	−0.004 (4)	0.075 (3)	−0.037 (4)
C28	0.100 (3)	0.092 (3)	0.108 (3)	0.003 (3)	−0.027 (3)	0.011 (3)
O1	0.0767 (14)	0.0461 (12)	0.0482 (11)	−0.0025 (11)	0.0015 (10)	0.0051 (10)
O2	0.151 (3)	0.0613 (16)	0.0715 (15)	0.0074 (16)	−0.0174 (16)	0.0165 (14)
O3	0.0643 (13)	0.0393 (11)	0.0510 (11)	0.0043 (9)	0.0154 (10)	0.0052 (9)
O4	0.0872 (17)	0.0554 (13)	0.0571 (13)	−0.0183 (13)	−0.0033 (12)	0.0089 (11)
O5	0.113 (2)	0.0729 (17)	0.108 (2)	−0.0099 (18)	−0.0277 (17)	0.0170 (18)
O6	0.0830 (14)	0.0477 (12)	0.0474 (11)	−0.0045 (11)	0.0164 (10)	−0.0009 (10)

### Geometric parameters (Å, °)

**Table:**

C1—O2	1.195 (4)	C15—H15	0.9800
C1—O1	1.374 (4)	C16—H16A	0.9600
C1—C2	1.455 (5)	C16—H16B	0.9600
C2—C3	1.316 (4)	C16—H16C	0.9600
C2—H2	0.9300	C17—H17A	0.9600
C3—C4	1.500 (4)	C17—H17B	0.9600
C3—H3	0.9300	C17—H17C	0.9600
C4—O3	1.395 (3)	C18—H18A	0.9600
C4—O1	1.454 (3)	C18—H18B	0.9600
C4—C7	1.528 (4)	C18—H18C	0.9600
C5—O5	1.196 (4)	C19—O6	1.467 (3)
C5—O4	1.334 (4)	C19—C24	1.514 (4)
C5—C6	1.491 (5)	C19—C20	1.519 (4)
C6—C7	1.519 (4)	C19—H19	0.9800
C6—H6A	0.9700	C20—C25	1.516 (5)
C6—H6B	0.9700	C20—C21	1.528 (5)
C7—C8	1.533 (4)	C20—H20	0.9800
C7—H7	0.9800	C21—C22	1.517 (6)
C8—O6	1.375 (3)	C21—H21A	0.9700
C8—O4	1.453 (4)	C21—H21B	0.9700
C8—H8	0.9800	C22—C23	1.507 (5)
C9—O3	1.453 (3)	C22—H22A	0.9700
C9—C14	1.512 (4)	C22—H22B	0.9700
C9—C10	1.525 (4)	C23—C24	1.531 (5)
C9—H9	0.9800	C23—C28	1.545 (5)
C10—C15	1.525 (5)	C23—H23	0.9800
C10—C11	1.540 (5)	C24—H24A	0.9700
C10—H10	0.9800	C24—H24B	0.9700
C11—C12	1.507 (5)	C25—C27	1.524 (5)
C11—H11A	0.9700	C25—C26	1.528 (6)
C11—H11B	0.9700	C25—H25	0.9800
C12—C13	1.500 (5)	C26—H26A	0.9600
C12—H12A	0.9700	C26—H26B	0.9600
C12—H12B	0.9700	C26—H26C	0.9600
C13—C18	1.516 (5)	C27—H27A	0.9600
C13—C14	1.524 (5)	C27—H27B	0.9600
C13—H13	0.9800	C27—H27C	0.9600
C14—H14A	0.9700	C28—H28A	0.9600
C14—H14B	0.9700	C28—H28B	0.9600
C15—C17	1.516 (5)	C28—H28C	0.9600
C15—C16	1.524 (6)		
			
O2—C1—O1	121.6 (3)	H16A—C16—H16B	109.5
O2—C1—C2	130.1 (3)	C15—C16—H16C	109.5
O1—C1—C2	108.3 (3)	H16A—C16—H16C	109.5
C3—C2—C1	109.2 (3)	H16B—C16—H16C	109.5
C3—C2—H2	125.4	C15—C17—H17A	109.5
C1—C2—H2	125.4	C15—C17—H17B	109.5
C2—C3—C4	109.8 (3)	H17A—C17—H17B	109.5
C2—C3—H3	125.1	C15—C17—H17C	109.5
C4—C3—H3	125.1	H17A—C17—H17C	109.5
O3—C4—O1	110.4 (2)	H17B—C17—H17C	109.5
O3—C4—C3	114.5 (2)	C13—C18—H18A	109.5
O1—C4—C3	103.5 (2)	C13—C18—H18B	109.5
O3—C4—C7	105.9 (2)	H18A—C18—H18B	109.5
O1—C4—C7	107.8 (2)	C13—C18—H18C	109.5
C3—C4—C7	114.7 (2)	H18A—C18—H18C	109.5
O5—C5—O4	121.6 (3)	H18B—C18—H18C	109.5
O5—C5—C6	127.8 (4)	O6—C19—C24	111.1 (2)
O4—C5—C6	110.6 (3)	O6—C19—C20	106.4 (2)
C5—C6—C7	105.8 (3)	C24—C19—C20	112.4 (3)
C5—C6—H6A	110.6	O6—C19—H19	108.9
C7—C6—H6A	110.6	C24—C19—H19	108.9
C5—C6—H6B	110.6	C20—C19—H19	108.9
C7—C6—H6B	110.6	C19—C20—C25	113.3 (3)
H6A—C6—H6B	108.7	C19—C20—C21	108.6 (3)
C6—C7—C4	113.6 (3)	C25—C20—C21	114.5 (3)
C6—C7—C8	104.3 (3)	C19—C20—H20	106.6
C4—C7—C8	111.7 (2)	C25—C20—H20	106.6
C6—C7—H7	109.0	C21—C20—H20	106.6
C4—C7—H7	109.0	C22—C21—C20	113.9 (3)
C8—C7—H7	109.0	C22—C21—H21A	108.8
O6—C8—O4	110.2 (2)	C20—C21—H21A	108.8
O6—C8—C7	109.5 (2)	C22—C21—H21B	108.8
O4—C8—C7	105.8 (2)	C20—C21—H21B	108.8
O6—C8—H8	110.4	H21A—C21—H21B	107.7
O4—C8—H8	110.4	C23—C22—C21	111.6 (3)
C7—C8—H8	110.4	C23—C22—H22A	109.3
O3—C9—C14	108.9 (2)	C21—C22—H22A	109.3
O3—C9—C10	107.1 (2)	C23—C22—H22B	109.3
C14—C9—C10	112.3 (2)	C21—C22—H22B	109.3
O3—C9—H9	109.5	H22A—C22—H22B	108.0
C14—C9—H9	109.5	C22—C23—C24	108.9 (3)
C10—C9—H9	109.5	C22—C23—C28	112.7 (3)
C9—C10—C15	114.2 (3)	C24—C23—C28	110.7 (3)
C9—C10—C11	109.0 (3)	C22—C23—H23	108.1
C15—C10—C11	114.6 (3)	C24—C23—H23	108.1
C9—C10—H10	106.1	C28—C23—H23	108.1
C15—C10—H10	106.1	C19—C24—C23	111.5 (3)
C11—C10—H10	106.1	C19—C24—H24A	109.3
C12—C11—C10	112.3 (3)	C23—C24—H24A	109.3
C12—C11—H11A	109.1	C19—C24—H24B	109.3
C10—C11—H11A	109.1	C23—C24—H24B	109.3
C12—C11—H11B	109.1	H24A—C24—H24B	108.0
C10—C11—H11B	109.1	C20—C25—C27	112.9 (4)
H11A—C11—H11B	107.9	C20—C25—C26	114.2 (4)
C13—C12—C11	112.5 (3)	C27—C25—C26	110.3 (4)
C13—C12—H12A	109.1	C20—C25—H25	106.3
C11—C12—H12A	109.1	C27—C25—H25	106.3
C13—C12—H12B	109.1	C26—C25—H25	106.3
C11—C12—H12B	109.1	C25—C26—H26A	109.5
H12A—C12—H12B	107.8	C25—C26—H26B	109.5
C12—C13—C18	112.8 (4)	H26A—C26—H26B	109.5
C12—C13—C14	109.2 (3)	C25—C26—H26C	109.5
C18—C13—C14	111.1 (3)	H26A—C26—H26C	109.5
C12—C13—H13	107.8	H26B—C26—H26C	109.5
C18—C13—H13	107.8	C25—C27—H27A	109.5
C14—C13—H13	107.8	C25—C27—H27B	109.5
C9—C14—C13	114.1 (3)	H27A—C27—H27B	109.5
C9—C14—H14A	108.7	C25—C27—H27C	109.5
C13—C14—H14A	108.7	H27A—C27—H27C	109.5
C9—C14—H14B	108.7	H27B—C27—H27C	109.5
C13—C14—H14B	108.7	C23—C28—H28A	109.5
H14A—C14—H14B	107.6	C23—C28—H28B	109.5
C17—C15—C10	110.9 (4)	H28A—C28—H28B	109.5
C17—C15—C16	109.6 (4)	C23—C28—H28C	109.5
C10—C15—C16	114.7 (4)	H28A—C28—H28C	109.5
C17—C15—H15	107.1	H28B—C28—H28C	109.5
C10—C15—H15	107.1	C1—O1—C4	109.1 (3)
C16—C15—H15	107.1	C4—O3—C9	119.1 (2)
C15—C16—H16A	109.5	C5—O4—C8	112.1 (3)
C15—C16—H16B	109.5	C8—O6—C19	114.9 (2)
			
O2—C1—C2—C3	−179.5 (4)	C11—C10—C15—C16	53.3 (4)
O1—C1—C2—C3	1.0 (4)	O6—C19—C20—C25	−55.8 (4)
C1—C2—C3—C4	−1.8 (4)	C24—C19—C20—C25	−177.7 (3)
C2—C3—C4—O3	122.0 (3)	O6—C19—C20—C21	175.7 (3)
C2—C3—C4—O1	1.8 (3)	C24—C19—C20—C21	53.8 (4)
C2—C3—C4—C7	−115.3 (3)	C19—C20—C21—C22	−52.8 (4)
O5—C5—C6—C7	176.8 (4)	C25—C20—C21—C22	179.4 (3)
O4—C5—C6—C7	−2.7 (4)	C20—C21—C22—C23	55.7 (4)
C5—C6—C7—C4	−112.9 (3)	C21—C22—C23—C24	−55.7 (4)
C5—C6—C7—C8	8.9 (3)	C21—C22—C23—C28	−178.9 (3)
O3—C4—C7—C6	63.9 (3)	O6—C19—C24—C23	−177.5 (2)
O1—C4—C7—C6	−177.9 (3)	C20—C19—C24—C23	−58.3 (4)
C3—C4—C7—C6	−63.2 (3)	C22—C23—C24—C19	57.5 (4)
O3—C4—C7—C8	−53.6 (3)	C28—C23—C24—C19	−178.1 (3)
O1—C4—C7—C8	64.5 (3)	C19—C20—C25—C27	173.2 (4)
C3—C4—C7—C8	179.2 (3)	C21—C20—C25—C27	−61.4 (5)
C6—C7—C8—O6	106.9 (3)	C19—C20—C25—C26	−59.7 (4)
C4—C7—C8—O6	−130.0 (3)	C21—C20—C25—C26	65.6 (4)
C6—C7—C8—O4	−11.8 (3)	O2—C1—O1—C4	−179.3 (3)
C4—C7—C8—O4	111.3 (3)	C2—C1—O1—C4	0.2 (3)
O3—C9—C10—C15	−58.6 (3)	O3—C4—O1—C1	−124.2 (3)
C14—C9—C10—C15	−178.1 (3)	C3—C4—O1—C1	−1.2 (3)
O3—C9—C10—C11	171.8 (3)	C7—C4—O1—C1	120.7 (3)
C14—C9—C10—C11	52.3 (4)	O1—C4—O3—C9	54.5 (3)
C9—C10—C11—C12	−54.9 (4)	C3—C4—O3—C9	−61.8 (3)
C15—C10—C11—C12	175.7 (3)	C7—C4—O3—C9	170.9 (2)
C10—C11—C12—C13	58.0 (5)	C14—C9—O3—C4	−93.9 (3)
C11—C12—C13—C18	−178.9 (4)	C10—C9—O3—C4	144.5 (2)
C11—C12—C13—C14	−54.8 (4)	O5—C5—O4—C8	175.2 (3)
O3—C9—C14—C13	−172.0 (3)	C6—C5—O4—C8	−5.3 (4)
C10—C9—C14—C13	−53.5 (4)	O6—C8—O4—C5	−107.3 (3)
C12—C13—C14—C9	53.1 (4)	C7—C8—O4—C5	11.0 (3)
C18—C13—C14—C9	178.2 (3)	O4—C8—O6—C19	−65.3 (3)
C9—C10—C15—C17	161.8 (4)	C7—C8—O6—C19	178.7 (2)
C11—C10—C15—C17	−71.5 (5)	C24—C19—O6—C8	−70.9 (3)
C9—C10—C15—C16	−73.5 (4)	C20—C19—O6—C8	166.4 (3)

### Hydrogen-bond geometry (Å, °)

**Table:**

*D*—H···*A*	*D*—H	H···*A*	*D*···*A*	*D*—H···*A*
C3—H3···O5^i^	0.93	2.37	3.289 (5)	168
C8—H8···O1	0.98	2.60	2.947 (4)	101
C14—H14A···O1	0.97	2.47	3.069 (4)	120
C15—H15···O3	0.98	2.47	2.915 (4)	107
C19—H19···O4	0.98	2.51	2.888 (4)	103
C25—H25···O6	0.98	2.45	2.853 (4)	105
C28—H28A···O2^ii^	0.96	2.59	3.405 (5)	143

Symmetry codes: (i) −*x*, *y*−1/2, −*z*+1; (ii) −*x*+1, *y*+1/2, −*z*+2.
